# An immunotherapy effect analysis in Rasmussen encephalitis

**DOI:** 10.1186/s12883-020-01932-9

**Published:** 2020-09-24

**Authors:** Zuzana Liba, Martina Vaskova, Josef Zamecnik, Jana Kayserova, Hana Nohejlova, Matyas Ebel, Jan Sanda, Gonzalo Alonso Ramos-Rivera, Klara Brozova, Petr Liby, Michal Tichy, Pavel Krsek

**Affiliations:** 1 Department of Pediatric Neurology, Second Faculty of Medicine, Charles University and Motol University Hospital, V Uvalu 84, 15006 Prague, Czech Republic; 2 CLIP - Childhood Leukaemia Investigation Prague, Department of Pediatric Haematology and Oncology, Second Faculty of Medicine, Charles University and Motol University Hospital, Prague, Czech Republic; 3 Department of Pathology and Molecular Medicine, Second Faculty of Medicine, Charles University and Motol University Hospital, Prague, Czech Republic; 4 Department of Immunology, Second Faculty of Medicine, Charles University and Motol University Hospital, Prague, Czech Republic; 5 Imunale s.r.o, Prague, Czech Republic; 6 Department of Neurology, Second Faculty of Medicine, Charles University and Motol University Hospital, Prague, Czech Republic; 7 Department of Radiology, Second Faculty of Medicine, Charles University and Motol University Hospital, Prague, Czech Republic; 8 Department of Pediatric Neurology, Comenius University Faculty of Medicine and National Institute of Children’s Diseases, Bratislava, Slovak Republic; 9 Department of Pediatric Neurology, Thomayer Hospital, Prague, Czech Republic; 10 Department of Neurology and Clinical Neuroscience, First Faculty of Medicine, Charles University and General University Hospital, Prague, Czech Republic; 11 Department of Neurosurgery, Second Faculty of Medicine, Charles University and Motol University Hospital, Prague, Czech Republic

**Keywords:** Rasmussen encephalitis, Immunotherapy effect, Chemokines, Cytokines, Lymphocyte subpopulations, Alemtuzumab, Intrathecal methotrexate

## Abstract

**Background:**

Immune-mediated mechanisms substantially contribute to the Rasmussen encephalitis (RE) pathology, but for unknown reasons, immunotherapy is generally ineffective in patients who have already developed intractable epilepsy; overall laboratory data regarding the effect of immunotherapy on patients with RE are limited. We analyzed multiple samples from seven differently treated children with RE and evaluated the effects of immunotherapies on neuroinflammation. Immunotherapy was introduced to all patients at the time of intractable epilepsy and they all had to undergo hemispherothomy.

**Methods:**

Immunohistochemistry, flow cytometry, Luminex multiplex bead and enzyme-linked immunosorbent assay techniques were combined to determine: 1) inflammatory changes and lymphocyte subpopulations in 45 brain tissues; 2) lymphocyte subpopulations and the levels of 12 chemokines/cytokines in 24 cerebrospinal fluid (CSF) samples and 30 blood samples; and 3) the dynamics of these parameters in four RE patients from whom multiple samples were collected.

**Results:**

Sustained T cell-targeted therapy with cyclophosphamide, natalizumab, alemtuzumab, and intrathecal methotrexate (ITMTX), but not with azathioprine, substantially reduced inflammation in brain tissues. Despite the therapy, the distributions of CD8^+^ T cells and the levels of C-X-C motif ligand (CXCL) 10, CXCL13, and B cell activating factor (BAFF) in patients’ CSF remained increased compared to controls. A therapeutic approach combining alemtuzumab and ITMTX was the most effective in producing simultaneous reductions in histopathological inflammatory findings and in the numbers of activated CD8^+^ T cells in the brain tissue, as well as in the overall CD8^+^ T cell population and chemokine/cytokine production in the CSF.

**Conclusions:**

We provide evidence that various T cell-targeted immunotherapies reduced inflammation in the brains of RE patients. The observation that intractable epilepsy persisted in all of the patients suggests a relative independence of seizure activity on the presence of T cells in the brain later in the disease course. Thus, new therapeutic targets must be identified. CXCL10, CXCL13 and BAFF levels were substantially increased in CSF from all patients and their significance in RE pathology remains to be addressed.

## Background

Rasmussen encephalitis (RE) is a rare chronic brain disorder characterized by progressive unihemispheric atrophy with a decline in hemispheric function and intractable epilepsy. Therapeutic management of RE remains controversial, and surgery is the only cure for the seizures caused by this disease [1].

The primary etiology of RE remains elusive, but immune-mediated mechanisms contribute to its pathology [1]. Immunohistochemical studies have revealed a complex engagement of T cells in the brain and a granzyme B-mediated attack on neurons and astrocytes, but the target antigens have not yet been identified [2, 3]. Both CD4^+^ and CD8^+^ T cell clones have been shown to produce multifunctional cytokines [3]. CD8^+^ T cell receptor sequencing revealed that T cell clones found in the brain are also expanded in the periphery and persist for years; clonal expansions in the CNS are the prominent feature of RE [4, 5].

Some positive effects of long-term immunotherapy with steroids, intravenous immunoglobulins (IVIG) or T cell-inactivating drugs (tacrolimus [TAC], azathioprine [AZA], cyclophosphamide [CPA]) have been observed in case reports or small patient series; none of these drugs has been shown to be superior or to halt intractable epilepsy [6–9]*.* Multiple sclerosis (MS) treatment studies have suggested novel candidate drugs for RE, such as rituximab (RTX), natalizumab (NAT) and alemtuzumab (ALEM), but clinical and laboratory data on their use in RE are limited [10–13].

We analyzed different samples from seven individually treated RE patients, who all underwent hemispherotomy due to intractable epilepsy, to evaluate the effect of immunotherapy on pathological findings in their brain tissues and on immune parameters in cerebrospinal fluid (CSF) and blood.

## Methods

### Ethics statement

The Ethics Committee at Motol University Hospital approved this study. Informed written consent to participate in the study was obtained from the parents of all pediatric participants.

### Study design

We evaluated neuroinflammatory and neurodegenerative changes in brain tissues from RE patients in relation to their immunotherapy. We compared lymphocyte subpopulations and the levels of twelve chemokines and cytokines in CSF and blood between patients and controls. The dynamics of the investigated parameters were assessed in four patients, who had multiple CSF and blood samples.

### Patients, clinical data and sampling

**Fig. 1:**
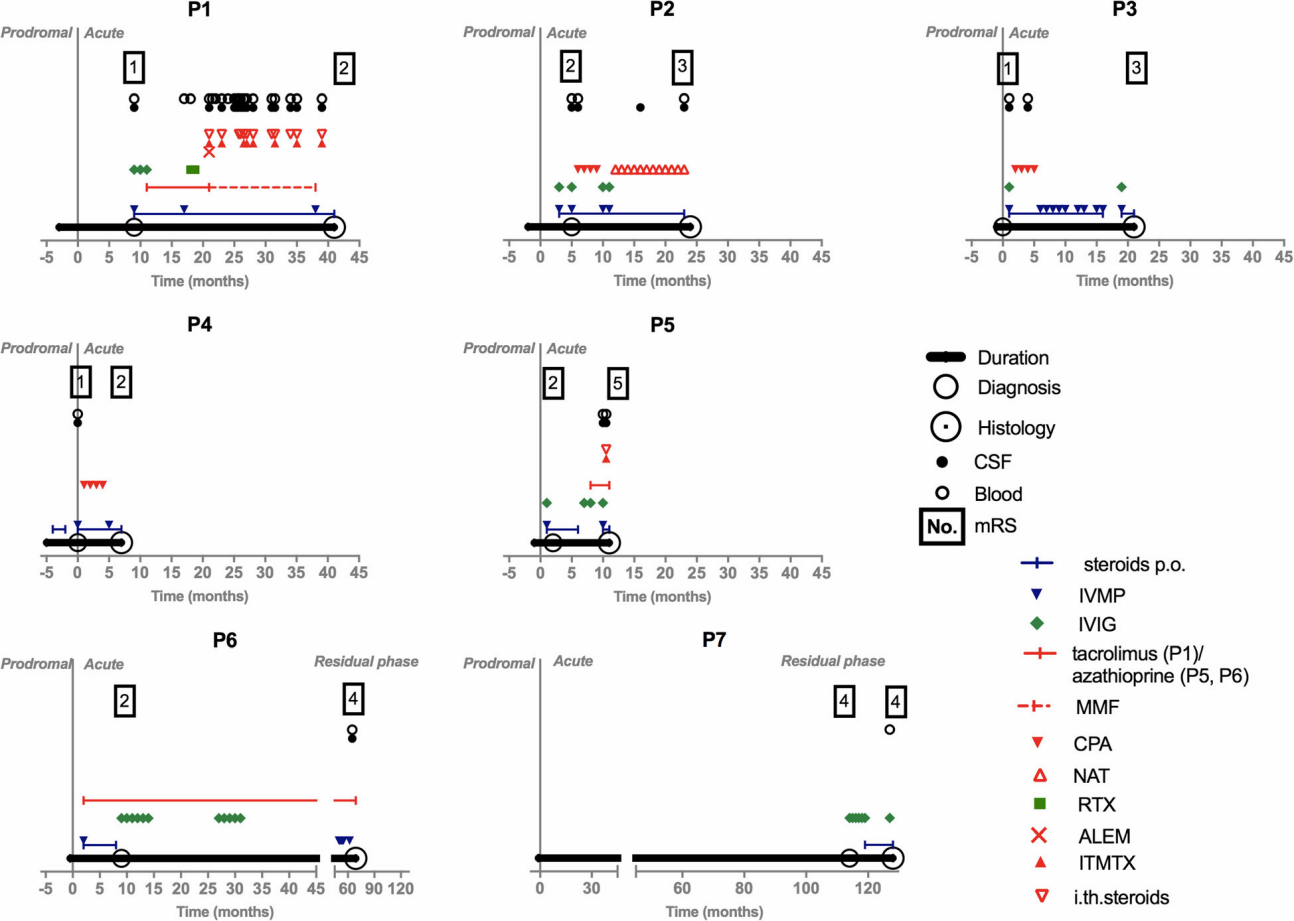
Sampling in the context of disease duration and immunotherapy. Disease duration defined the interval from the appearance of the first symptom until functional hemispherotomy in every patient; the correct diagnosis was made after a delay from the first symptom. The prodromal phase of the disease characterized infrequent seizures and no hemiparesis. The seizure frequency strikingly increased, all patients developed intractable epilepsy, and an individual functional decline in the affected hemisphere and its atrophy became evident in an acute phase of the disease. The time of a transition to the residual phase was not clearly clinically recognizable, and all patients suffered from intractable epilepsy until neurosurgery. The clinical outcome in every patient at the time of disease diagnosis and then at the time of neurosurgery is displayed using modified Rankin scale (mRS); the numbers in squares express mRS scores. In addition, immunotherapies are shown, and the type and time of the sample withdrawals are noted in every patient

Seven patients with intractable epilepsy (median age 7 years, range 3–15; 71% females) who were referred to the Motol Epilepsy Center, Prague, Czech Republic, in 2012–2018 and fulfilled Bien’s 2005 diagnostic criteria for RE [14] were included in this study. Clinical data, focused on disease onset and immunotherapy, were collected until neurosurgery. In total, 24 CSF samples and 30 blood samples were obtained from these patients during the diagnostic process and therapeutic management; the majority were drawn during immunotherapy (22/24 CSF, 28/30 blood) and from patient 1 (P1; 14/24 CSF, 20/30 blood). All patients underwent functional peri-insular hemispherotomy as described by Villemure [15]; brain tissue samples from different parts of the affected hemisphere were taken for immunohistochemistry (45/45) and flow cytometry (28/45). For details on sampling, see Fig. 1 and Additional file 1.

### Controls

Eight patients with other noninflammatory neurological disorders were selected as controls (median age 12 years, range 11–17; 50% females). These patients underwent lumbar taps and blood testing during the routine diagnostic process. Flow cytometry was performed in five of these controls, but brain tissues were not available. The final diagnoses (based on genetics and/or MRI findings) were as follows: Alexander disease, gliomatosis cerebri, Leber hereditary optic neuropathy, tuberous sclerosis, oculomotor nerve neurinoma, and brainstem tumor (*n* = 3).

### Chemokine and cytokine detection in blood and CSF via Luminex multiplex bead technology and enzyme-linked immunosorbent assay (ELISA)

Aliquots of centrifuged CSF and serum were immediately stored at − 30 °C and thawed prior to use for chemokine/cytokine analyses. The concentrations of C-C and C-X-C motif chemokines (CCL2, CXCL8, CXCL10, CXCL13) and cytokines (interleukin [IL]-4, IL-6, IL-7, IL-10, IL-15, IL-17A, interferon gamma [IFN-γ]) were measured. We combined multiple simplex kits with a basic kit according to the manufacturer’s instructions (Human ProcartaPlex Simplex Kits and ProcartaPlex Human Basic Kit, Thermo Fisher Scientific/formerly eBioscience, San Diego, CA, USA). The methodological details, including the assay protocol, standards and sensitivity, are available at the manufacturer’s website, http://www.thermofisher.com. The data were collected using a Luminex-100 system (Luminex, Austin, TX, USA). In addition, B cell-activating factor (BAFF) concentrations were determined via ELISA (Human BAFF/BLyS/TNFSF13B Quantikine Kit, R&D Systems, Minneapolis, MN, USA) according to the manufacturer’s instructions using software from R&D Systems.

### Determination of lymphocyte subpopulation distributions in blood, CSF and brain tissue via flow cytometry

Brain tissues were immediately minced with a lancet, shaken for 30 min on a petri dish in a phosphate-buffered saline solution with 2 mM ethylenediaminetetraacetic acid to prevent aggregation, filtered through a 100 μm cell strainer (Biologix Group Limited, Shandong, China) and pelleted by centrifugation. Blood and CSF samples were immediately processed for antibody staining according to the routine protocols. Lymphocyte subpopulations were evaluated using the following antibody mixtures: 1) CD3 FITC, HLADR PE, CD45 PerCP, CD4 PE-Cy7, CD19 APC, CD8 APC-Cy7, CD14 PB and 2) CD3 FITC, CD16 + CD56 PE, CD45 PerCP-Cy5.5, CD4 PE-Cy7, CD19 APC, CD8 APC-Cy7, HLADR PB (both Exbio, Prague, Czech Republic). Samples were measured with one of the following flow cytometers: BD LSRII, BD FACSLyric (BD Biosciences, San Jose, CA, USA), Cyan ADP Flow Cytometer (Dako, Glostrup, Denmark). The data were analyzed in FlowJo software, version 8.8.7 (FlowJo, LLC, Ashland, OR). The distributions of CD19^+^, CD3^+^, CD4^+^ and CD8^+^ cells are expressed as percentages from the lymphocytic gate (CD45^++^ cells and the side scatter corresponding to lymphocytes), and activation is expressed as a percentage of HLADR^+^ cells among CD4^+^ or CD8^+^ T cells (HLADR^+^/CD3^+^CD4^+^, HLADR^+^/CD3^+^CD8^+^).

### Histology and immunohistochemistry

Resected tissues were fixed in 10% buffered formalin and embedded in paraffin. In each case, a search for gliosis and lymphocytes in the serial cryosections was performed using both a routine hematoxylin and eosin (H&E) stain and immunohistochemical reactions with anti-GFAP (clone 6F2, Dako, diluted 1:1000) and anti-CD45 (leukocyte common antigen - LCA, clone 2B11 + PD7/26, Dako, diluted 1:100) antibodies, respectively. Provided that lymphocytes were found, the contiguous sections were subjected to immunohistochemistry to establish their phenotype using antibodies against the lymphocytic surface antigens CD20 (clone L26, Dako, diluted 1:300), CD8 (clone C8/144B, Dako, diluted 1:200) and CD4 (clone 4B12, Novocastra, diluted 1:20). The antigen-antibody complexes were visualized with biotin-streptavidin detection systems (N-Histofine Simple Stain MAX PO, Nichirei Corporation, Tokyo, Japan); chromogenic development was performed using 3,3-diaminobenzidine. All sections were counterstained slightly with Harris’ hematoxylin. Positive and negative controls were used in each assay.

### Data visualization and statistics

GraphPad Prism software version 6.0 (La Jolla, CA, USA) was used to create graphs and perform statistical analysis. Due to the nature of the data, nonparametric tests were used; the Kruskal-Wallis test with a post hoc Dunn’s test were used for comparisons between multiple groups and the Mann-Whitney U test was employed for unpaired comparisons between two groups. *P* < 0.05 was considered statistically significant.

## Results

### Clinical data

**Fig. 2:**
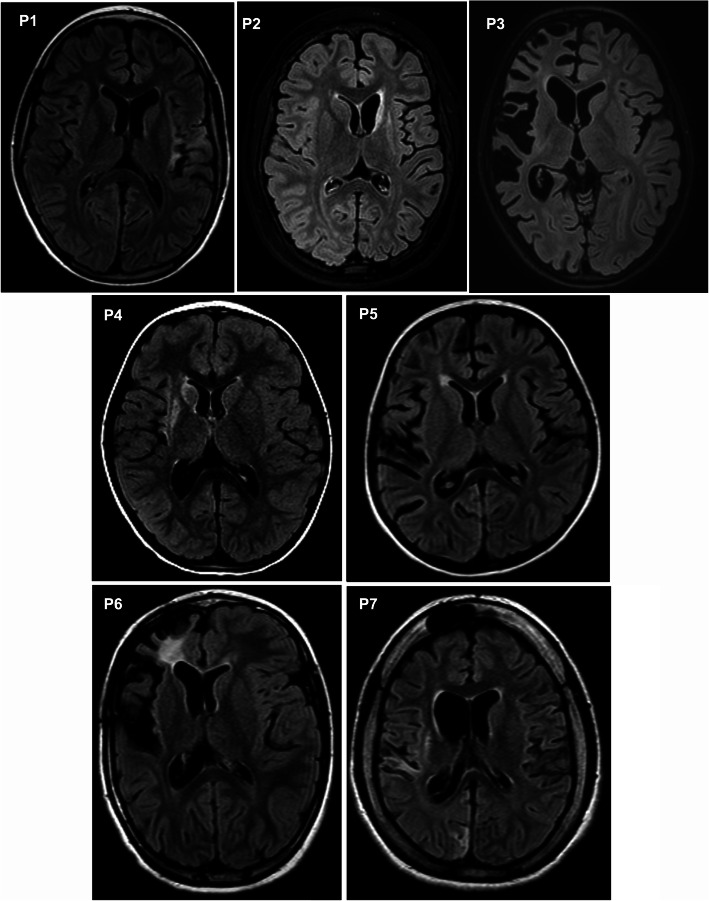
Magnetic resonance imaging (MRI) at the time of surgery. Axial fluid-attenuated inversion recovery (FLAIR) MRI sequences of the brain showing differently pronounced atrophy of the affected hemisphere in every patient at the time of surgery are displayed. In addition, various inflammatory and glial changes in the affected hemisphere can be observed in every patient

All patients were children with normal psychomotor development until RE onset; in all of them, RE began with a focal epileptic seizure of variable semiology (median age 4 years, range 2–9; 71% females). After a variable period (median 1 month, range 0.5–5), all children developed intractable epilepsy followed by individual motor and cognitive decline. The correct diagnosis was made after a delay from first disease symptom onset (median 9 months; range 1–115). Immunotherapy was introduced at the time of intractable epilepsy in all cases; the exception was P4, who received a low dose of oral steroid in a prodromal phase due to an abnormal brain imaging. In addition, P2 and P6 received immunotherapy prior the correct diagnosis because they had other clinical symptoms (cerebellar syndrome in P6) and abnormal brain imaging (P2) suspected from brain inflammation. The first choice for all was intravenous methylprednisolone (IVMP) with an oral taper and/or IVIG. Subsequent T cell-targeted immunotherapy was introduced in P1–6 (i.e., AZA, TAC or intravenous CPA). Therapy was escalated further in the two patients with the dominant hemisphere affected. P1 received one cycle of RTX after 9 months of unsatisfactory therapy with TAC and then one cycle of ALEM that was combined with intrathecal methotrexate (ITMTX) in irregular intervals and long-term mycophenolate mofetil treatment (MMF) [13]. P2 was escalated to NAT after four doses of intravenous CPA. None of the therapies reversed the refractory epilepsy, although all patients received some temporary positive impact on their clinical state. In P1 alone, therapeutic management stopped the motor (not cognitive) decline and modified the course of intractable epilepsy (periods of relative seizure stabilization alternated clusters of seizures) [13]. Hemispherotomy was finally performed in all of the children (median 26 months after disease onset, range 12–126). An initial disconnection was incomplete in four patients; they thus were re-operated (P2, P4, P5 and P7). All but one patient (P7: the one with the longest disease duration and minimal immunotherapy) remained seizures free after a complete disconnection of the affected hemisphere. The clinical data are summarized in Fig. 1 and Additional file 1. The individual atrophy of the affected hemisphere at the time of neurosurgery is captured in Fig. 2.

### Laboratory data

#### Brain tissues

Histopathological findings at the time of hemispherotomy were consistent with the diagnosis of RE in every patient. Although the neuroinflammatory and neurodegenerative changes varied in the patients, three distinct patterns were identified: A) In P1, P2 and P4 – low inflammatory activity with scarce lymphocytes, no neuronophagy and glial nodules, mild gliosis, and minimal cortical atrophy. B) In P3 and P5 – inflammatory activity with dense lymphocytic infiltrates, neuronophagy, satellitosis, glial nodules, severe gliosis, and cortical atrophy. C) In P6 and P7 – mild inflammatory activity with isolated lymphocytes, sporadic satellitosis and glial nodules, medium to severe gliosis, and diffuse cortical atrophy.

**Fig. 3:**
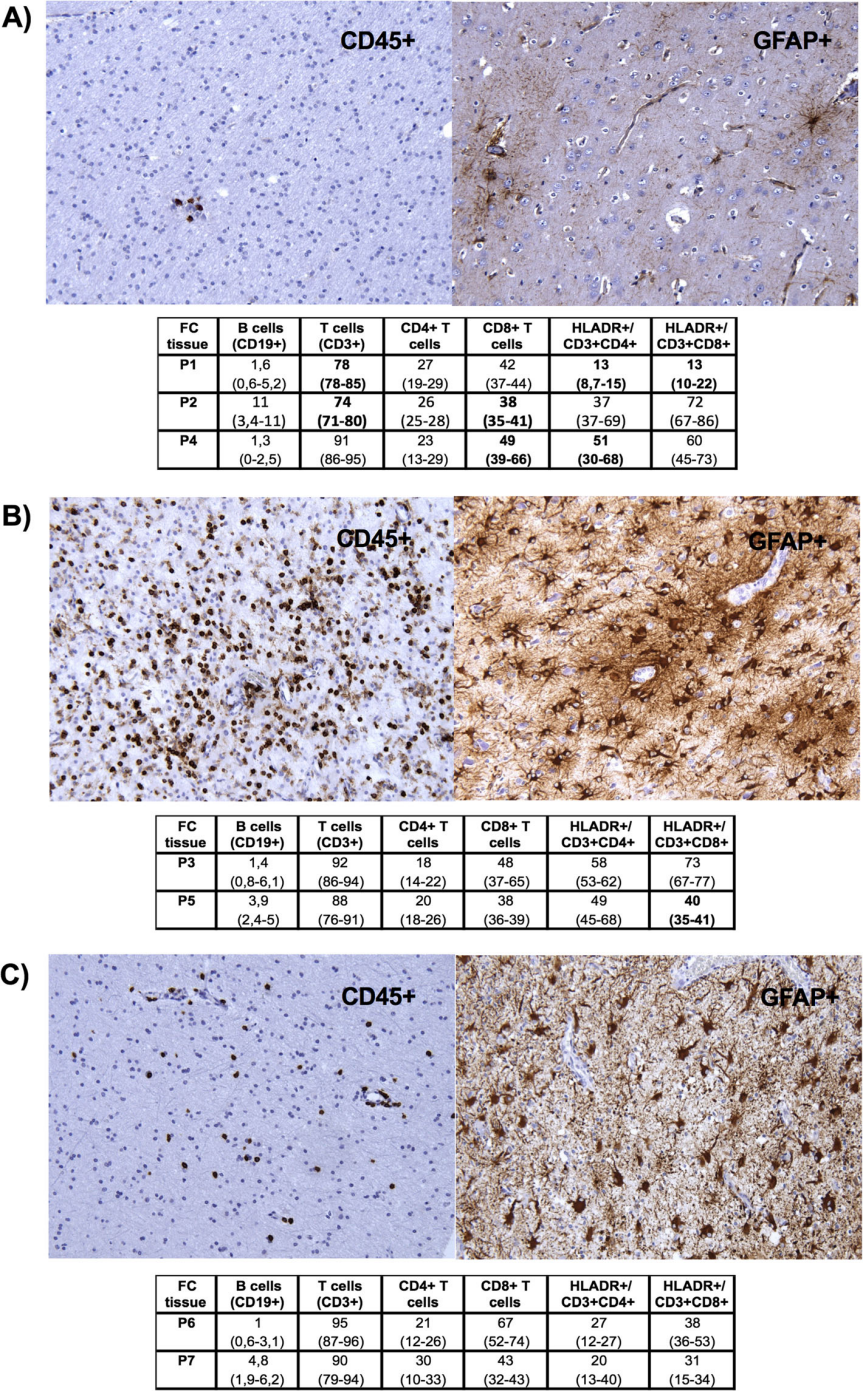
Immunohistochemistry and flow cytometry of the brain tissues from differently treated patients with RE. Three multipanels show a representative pair of histopathological findings (magnification × 200) according to the different immunohistochemical staining and a table that summarized lymphocyte subpopulations in the brain tissues from patients corresponding to the histopathology. Immunostaining for CD45 (anti-CD45, leukocyte common antigen) identifies leukocytes in the brain tissue, and flow cytometry (FC) further determines their type. The distribution of CD19^+^, CD3^+^, CD4^+^ and CD8^+^ cells is expressed as a percentage from the lymphocytic gate (CD45^++^ cells and the side scatter corresponding to lymphocytes) and the activation as a percentage of HLADR^+^ cells from CD4^+^ or CD8^+^ T cells (HLADR^+^/CD3^+^CD4^+^, HLADR^+^/CD3^+^CD8^+^); several brain samples were measured in every patient and the median values and ranges are displayed. Immunoreactivity for astrocytic glial fibrillary acidic protein (anti-GFAP) reveals gliosis. **a** Low neuroinflammatory activity with scarce inflammatory cells and mild gliosis were found in P1, P2 and P4. Despite the same histopathological pattern, significant differences in CD3^+^, CD8^+^, HLADR^+^/CD3^+^CD4^+^ and HLADR^+^/CD3^+^CD8^+^ lymphocyte subpopulations were identified among these patients (Kruskal-Wallis and Dunn’s test in a post hoc analysis were employed; numbers in bold; details in the text). **b** High neuroinflammatory activity with lymphocytic infiltrates and severe gliosis was found in P3 and P5; a significantly lower percentage of HLADR^+^/CD3^+^CD8^+^ in the brain tissue of P5 was identified (Mann-Whitney test). **c** Medium neuroinflammatory activity with isolated inflammatory cells and medium to severe gliosis in the brain tissue exerted P6 and P7; no significant differences in lymphocyte subpopulations were found (Mann-Whitney test)

Flow cytometry of brain tissues confirmed that the majority of infiltrating lymphocytes were T cells with a CD8^+^ phenotype and different activation states; CD4^+^ T cells and a few CD19^+^ B cells were also detected in the tissues. Interpretations of patient-by-patient differences in lymphocyte subpopulations were related to the corresponding histopathological pattern. Among the three patients (P1, P2, P4) with minimal inflammatory changes, P1 had the lowest percentage of activated T cell subpopulations; HLADR^+^/CD3^+^CD8^+^ cells were lower in P1 than in the other two patients (*P* < 0.04), but the HLADR^+^/CD3^+^CD4^+^ cells were decreased only compared with P4 (*P* < 0.01). In addition, P1 and P2 had lower percentages of overall CD3^+^ and CD8^+^ T cells than P4; statistical significance for CD8^+^ cells was reached only between P2 and P4 (P < 0.04 for all). In the comparison of samples with high inflammatory activity from P3 and P5, we observed a lower percentage of HLADR^+^/CD3^+^CD8^+^ cells in P5 (*P* = 0.0357). No significant differences in lymphocyte subpopulations were identified between brain tissues from P6 and P7 (Fig. 3).

#### CSF and blood

**Fig. 4:**
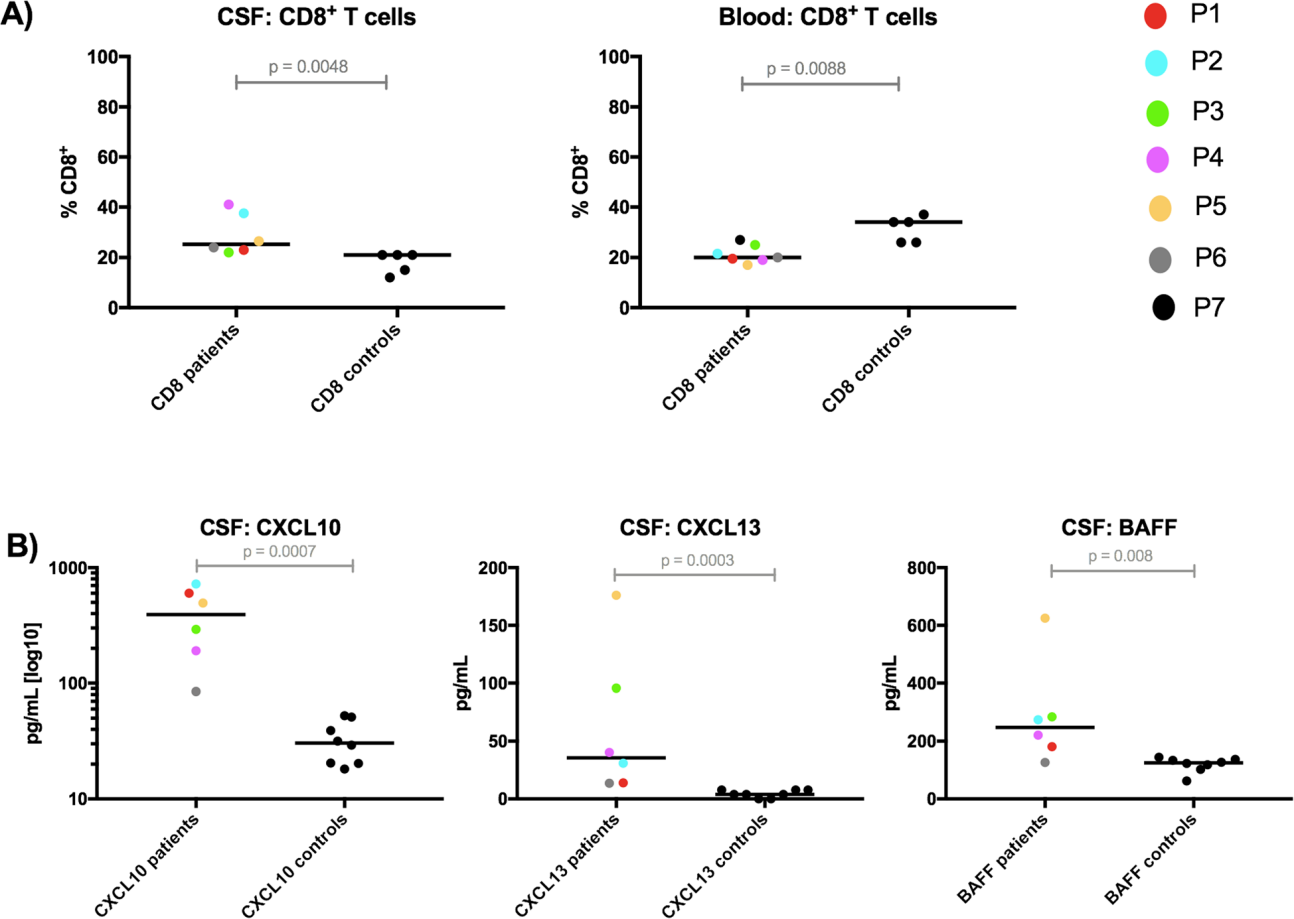
Lymphocyte subpopulations and chemokine/cytokine levels from treated RE patients’ group in comparison with controls. Parameters with significant differences are displayed: **a** Contrast between the overall CD8^+^ T cell subpopulation in CSF and blood (% expressed from lymphocytes). **b** High levels of CXC10, CXCL13 and BAFF in CSF. The Mann-Whitney test was employed for the comparisons of the investigated parameters in treated RE patients with the controls; the median values were used for patients with multiple samples (P1, P2, P5). The patients are indicated with different-colored dots; no CSF sample from P7 was available

Lymphocyte subpopulations and chemokine/cytokine levels were compared in samples collected after initiation of immunotherapy with the controls to identify persisting differences; the medians were used for the patients with > 1 sample (Fig. 4). The absolute immune cell numbers in CSF are not displayed, as they were very low in all analyzed samples (i.e., < 3 cells/μL). In addition, we analyzed only those chemokine/cytokines, whose levels were above the detection limits in the majority of the CSF and blood samples (i.e., CXCL8, CXCL10, CXCL13, CCL2, IL-7 and BAFF in both fluids; IL-10, IL-17A and IFN-γ in blood).

In contrast to the brain tissue, we observed T cells with a CD4^+^ phenotype predominance and no CD19^+^ B cells in the majority of CSF samples from treated patients and controls; if activated, CD8^+^ > CD4^+^ T cells. An overall CD8^+^ T cell population (not the percentage of HLADR^+^/CD3^+^CD8^+^) was higher in patients’ CSF samples (*P* = 0.0048). Regarding chemokine/cytokine levels, CXCL10, CXCL13 and BAFF were increased in CSF samples from treated patients compared with the controls (*P* < 0.008 for all).

At the same time, an overall CD8^+^ T cell population (not the percentage of HLADR^+^/CD3^+^CD8^+^) in blood from treated patients was lower in comparison to the controls (*P* = 0.0088), and no statistically significant differences for all analyzed chemokine/cytokine levels were identified.

#### Individual dynamics of the investigated parameters

**Fig. 5:**
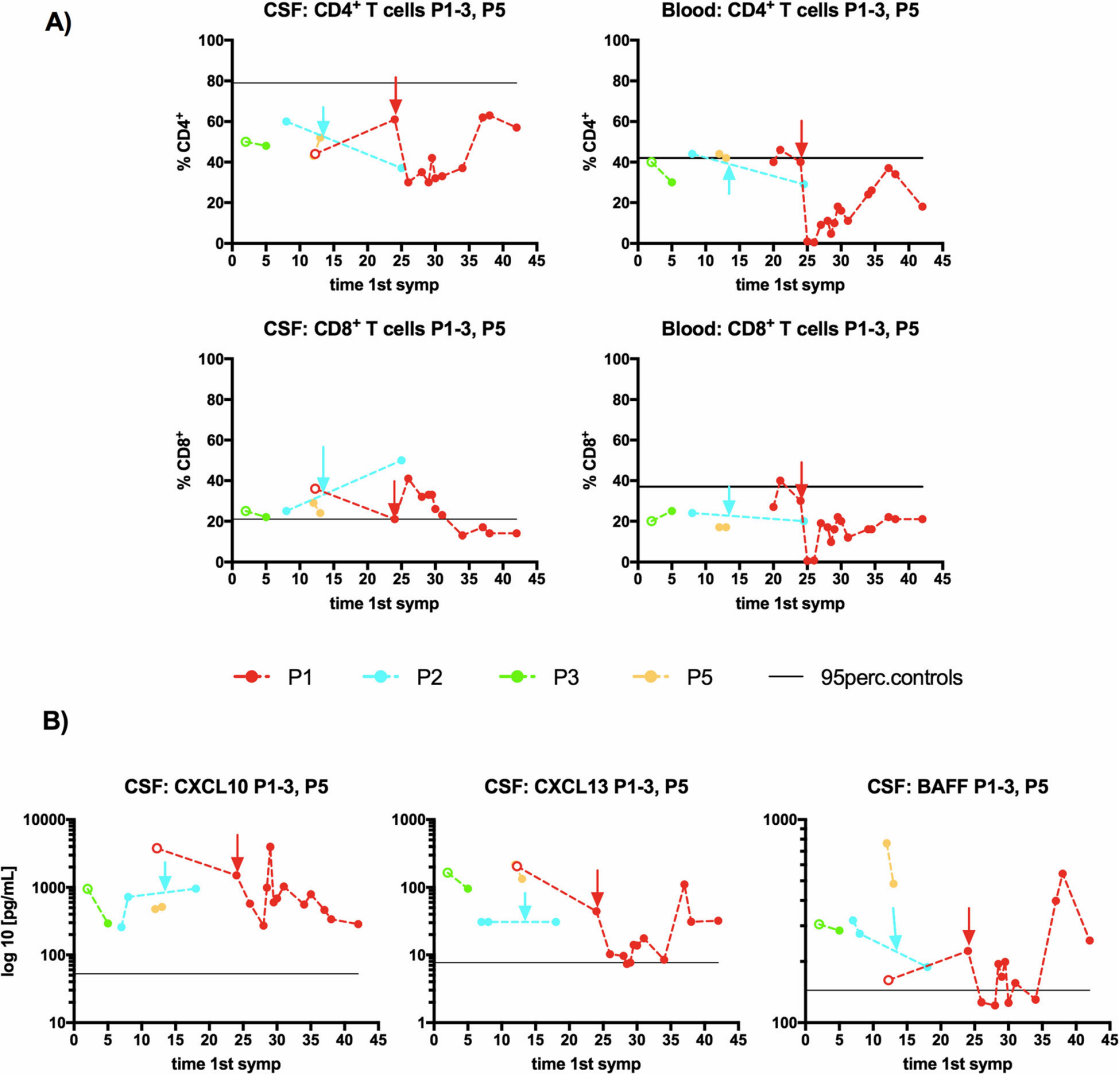
Individual dynamic changes of selected parameters in the CSF and blood during treatment. Dynamic changes in the levels of selected parameters from P1–3 and P5 are displayed in the context of the disease duration (since the first disease symptom appeared). **a** Different effects of immunotherapy on the CD4^+^ and CD8^+^ subpopulations behind the blood-brain barrier and in blood are documented (% expressed from lymphocytes). **b** Persistently increased levels of CXCL10, CXCL13, and BAFF in the CSF (above the 95th percentile of the controls) are depicted; the level of BAFF temporarily decreased under the 95th percentile of the controls only in P1 after ALEM administration, and during that period, CXCL13 level and CD4^+^ subpopulation in the CSF were also reduced. The patients are indicated with different colors. The empty dots represent samples collected prior to any immunotherapy and the arrows indicate ALEM administration in P1 and the initiation of NAT treatment in P2

Finally, we separately analyzed those samples that were obtained from P1–3 and P5 at different time points of their disease and in relation to their therapy. Of all the investigated parameters, we especially focused on the dynamics of those parameters showing differences between patients with RE and controls in the analyses described above (i.e., CD8^+^ T cell subpopulations, and CXCL10, CXCL13 and BAFF, Fig. 5 and Additional file 2).

##### P1

In contrast to the immediate depletion of CD4^+^ and CD8^+^ T cell subpopulations in the blood after ALEM administration (1–2%), these subpopulations were still detectable in the CSF. Interestingly, the percentage of CD8^+^ T cells in CSF even increased after ALEM (41%) and then continuously decreased to its minimum until ALEM posttreatment (APT) month 10 (13%) and remained low. The CD4^+^/CD8^+^ ratio in CSF permanently remained > 1, except for the first after ALEM. In contrast to the CSF, CD8^+^ T cells in blood had repopulated in APT month 13 and they repopulated faster than CD4^+^ T cells (APT months 6 vs. 13), which temporarily shifted the CD4^+^/CD8^+^ ratio to < 1. In addition, different changes in chemokine/cytokine levels were observed in the blood and CSF during the APT period. Notably, CXCL10, CXCL13 and BAFF levels in the CSF were initially all above the 95th percentile of the controls, and although CXCL10 and CXCL13 production in the CSF was strikingly decreased after RTX and ALEM administration, the levels still remained above that percentile. The level of BAFF in the CSF was, markedly decreased after ALEM, and similar to CXCL13, the levels increased in APT month 13. The dynamic changes in CXCL10, CXCL13 and BAFF levels in the blood are captured in Additional file 2.

##### P2

After 12 doses of NAT (CPA preceded NAT), a reversal of the CD4^+^/CD8^+^ ratio was detected in the CSF sample compared with the sample collected during steroid and IVIG therapy (0.74 vs. 2.4). CXCL10, CXCL13, and BAFF levels in the CSF were above the 95th percentile of the controls, and the chemokines further increased upon NAT treatment.

##### P3 and P5

After three doses of CPA (P3) and immediately after IVMP and IVIG (P5), a reduction in the percentage of CD8^+^ T cells was observed in the CSF. CXCL10, CXCL13 and BAFF levels in the CSF decreased but remained above the 95th percentile of the controls.

## Discussion

The study provides unique immunological data from patients with RE who were treated with potent and promising immunotherapy, such as RTX, NAT or ALEM. Because RE is a very rare disease with an estimated incidence of 2–3 cases per 10^7^ [1], we analyzed all available material.

We clearly demonstrated that patients with different therapeutic approaches and timing of neurosurgery exerted differently pronounced inflammatory and degenerative changes in a brain tissue; substantial reductions in both aspects was observed in three patients (P1, P2, P4) only with sustained T cell-targeted therapy (CPA, NAT or ALEM). In contrast, the most pronounced pathological changes were in the patients who discontinued this therapy with CPA 15 months prior to hemispherotomy (P3) and who had long-term treatment with AZA (P5). Neuroinflammation naturally decreases over time in untreated individuals with RE [16, 17]. Thus, the effect of immunotherapy was difficult to evaluate in two patients with the longest disease symptoms duration (P6, P7), whose histopathological findings in the brain tissue corresponded to the residual phase of the disease.

Determinations of lymphocyte subpopulations in the brain tissues (especially T cell subtypes and their states of activation) enabled us to better assess the effects of individual therapeutic approaches. Thus, despite the similarity of the histopathological pattern in the brains of three patients (P1, P2 and P4) with significantly reduced signs of neuroinflammation, we were able to determine that patients treated with ALEM and ITMTX (P1) and NAT (P2) had lower percentages of cytotoxic T cells than patient treated with CPA (P4); the patient treated with ALEM and ITMTX (P1) had the lowest percentage of activated cytotoxic T cell as well. A low percentage of activated cytotoxic T cells was also determined in another patient receiving ITMTX prior to neurosurgery (P5), who had otherwise prominent inflammation in histopathology. This suggests effect of ITMTX on activated cytotoxic T cell subpopulation. ITMTX is commonly used in patients with leukemia to reduce the risk of CNS relapse [18] and was shown to be beneficial for some progressive MS cases [19].

Monitoring lymphocyte subpopulations and chemokine/cytokine levels in CSF and blood gave us additional insight into the treatment effect. The treated patients’ group showed a discrepancy between the increased CD8^+^ T cell subpopulation in CSF and its reduction in blood and persistently increased levels of CXCL10, CXCL13, and BAFF in CSF compared with the controls. We assume that this may reflect a poor clinical response and immunotherapy failure in the CNS compartment.

However, detailed analyses of lymphocyte subpopulations in individuals with multiple samples showed reductions in the CD8^+^ T cell populations in the CSF of all patients receiving immunotherapy, except the one treated with NAT (P2). Interestingly, only in the patient treated with ALEM and ITMTX (P1) this population gradually decreased under the 95th percentile of the controls. Notably, our data from P1 provide new insight into the effect of ALEM behind the blood-brain barrier, while our findings from blood correspond to the effect of ALEM in MS patients [20–22]. In addition, only in P2 immunotherapy reversed the CD4^+^/CD8^+^ ratio in the CSF to < 1, which has already been described in MS patients treated with NAT, as the T cells are trapped in the periphery [23].

Moreover, although the levels of CXCL10, CXCL13, and BAFF in the CSF tended to decrease during therapy, they generally remained above the 95th percentile of the controls. This finding is interesting because CXCL10 and CXCL13 levels are frequently increased in many patients with neuroinflammatory conditions and play prominent roles in maintaining of intrathecal inflammation [24–30]. CXCL13 is a potent B cell chemoattractant [25, 26] and we previously reported its high specificity and sensitivity as a marker of neuroinflammation with different origins [27]. We were unable to clearly distinguish between the effect of the immunotherapy on chemokine/cytokine levels and a natural course of the pathology. The lowest concentrations of CXCL10, CXCL13 and BAFF were in the patient in a residual phase of the disease (P6). However, we observed in the CSF of P1 temporal reduction in CXCL13 and BAFF, which was related to the period of low CD4^+^ T cell population after ALEM treatment. In addition, we could see continuous decrease in CXCL10 along with continuous reduction in CD8^+^ T cells in the CSF of P1 during therapy, and we hypothesized that all these findings might reflect some decrease in inflammatory activity. CXCL10 binds to C-X-C receptor 3 (CXCR3), which is highly expressed on T cells and recruits them to the site of inflammation. In addition to chemoattraction, the CXCL10-CXCR3 axis has other immunological functions [28–30]. CD8^+^ T cells in the brain tissue from patients with RE express CXCR3, whereas neurons and astrocytes in the same areas express CXCL10 [31]. We observed strikingly higher CXCL10 levels in all patients. However, the patient treated with NAT (P2) was the only one in whom the level did not decrease during therapy. In addition, the finding from P2 contrasts with that in MS patients receiving NAT treatment [32]. According to a recent study, NAT enhances the inflammatory functions of T cells with residual migratory capacity, namely, by increasing the production of IFN-γ [33]. Interestingly, IFN-γ (alone or in combination with IL-1β and tumor necrosis factor [TNF]-α) is a capable inducer of CXCL10 production [28]. Regarding this aspect and the change in the CD4^+^/CD8^+^ ratio in favor of CD8^+^ T cells in the CSF of the patient treated with NAT, the potential benefit of this therapy in RE patients should be carefully considered. In addition, we determined high levels of BAFF in this study. BAFF is crucial for B cell survival and activation and influences proinflammatory T cell functions [34]. The substantial roles of B cells in T cell-driven autoimmunity, such as MS, are already well known [35]. However, there are very limited data about B cells in RE patients in addition to their very low distribution in brain infiltrates [3].

Taken together, sustainable and aggressive T cells-targeted immunotherapy reduced inflammation in the brains of RE patients. The therapeutic approach including ALEM and ITMTX was the most effective but with serious adverse reaction after ALEM [13]. Although being aware of our limited and heterogeneous study group, the observation that intractable epilepsy persisted in our patients despite a significant decrease in T cells suggests a relative independence of seizure activity on the presence of T cells in the brain tissue later in the disease course. More studies are needed to clarify if the aggressive T-cell-targeted immunotherapy early in the disease course is capable to prevent further RE progression and intractable epilepsy [36]. Our results are consistent with previous clinical observations, in which immunotherapy appeared to slow the functional decline rather than affect the seizure activity in the patients who had already developed intractable epilepsy [7, 9, 11]. A recent study using an animal model further supported these observations [37]. However, Bien’s 2005 diagnostic criteria, the lack of early disease-specific clinical symptoms and laboratory biomarkers usually unable clinicians to start immunotherapy before the disease is established in its acute phase with frequent seizures and ongoing brain atrophy.

Interestingly, our data show a persistent elevation of proinflammatory chemokines and BAFF in the CSF, suggesting that other possible mechanisms might be involved in RE pathology and should be explored along with chemokine/cytokine-targeting treatment options. Based on accumulating evidence, innate immunity is also generally involved in epileptogenesis, and together with adaptive immunity, it contributes to the maintenance of epileptic activity of different origins [38, 39]. Therefore, a pilot study with anti-TNF-α therapy in RE patients was conducted and produced promising results [40]. Hence, the constant search for therapeutic target other than T cells in RE patients must continue.

## Conclusion

We describe the effect of T cell-targeted therapy on neuroinflammation in RE patients. We provide evidence that reduced inflammation in the brains of RE patients did not halt intractable epilepsy once it had already developed. CXCL10, CXCL13 and BAFF levels were strikingly increased in the CSF from all patients, and thus their importance in RE pathology must be addressed.

## Supplementary information

**Additional file 1.** Summary of the clinical data, immunotherapy and sample investigations (additional_file_1.docx).

**Additional file 2.** Individual dynamic changes in chemokine/cytokine levels in the blood during treatment (additional_file_2.tiff). The dynamic changes in the levels of CXCL10, CXCL13 and BAFF levels in the blood are captured. Despite the dynamic changes, the levels of CXCL10 and CXCL13 in the blood generally did not exceed the 95th percentile of controls; this contrasted with the findings in CSF. In contrast, the blood BAFF level frequently exceeded the 95th percentile of controls. In P1, the BAFF level increased after RTX and ALEM treatment and decreased until month 18. In the remaining patients, BAFF levels tended to decrease after treatment.. The patients are indicated with different colors. The empty dots represent samples collected prior to any immunotherapy and the arrows indicate ALEM administration in P1. No blood sample was available from P2 for chemokine/cytokine analysis after the initiation of NAT treatment.

## Data Availability

All data analyzed during this study are included in this published article.

## Abbreviations

ALEM: Alemtuzumab
APT: Alemtuzumab posttreatment
AZA: Azathioprine
BAFF: B cell activating factor
CCL: C-C motif ligand
CD: Cluster of differentiation
CPA: Cyclophosphamide
CSF: Cerebrospinal fluid
CXCL: C-X-C motif ligand
HLA: Human leukocyte antigen
IFN-γ: Interferon gamma
IL: Interleukine
ITMTX: Intrathecal methotrexate
IVIG: Intravenous immunoglobulins
IVMP: Intravenous methylprednisolone
NAT: Natalizumab
RE: RASMUSSEN encephalitis
RTX: Rituximab
TAC: Tacrolimus
