# Supplementary material for: An immunotherapy effect analysis in Rasmussen encephalitis
Source: BMC Neurol. 2020 Sep 24;20:359. doi: 10.1186/s12883-020-01932-9 (PMC7517818; doi:10.1186/s12883-020-01932-9)
Supplement: Supplementary file 1 — Additional file 1. Summary of the clinical data, immunotherapy and sample investigations (additional_file_1.docx). [file 12883_2020_1932_MOESM1_ESM.docx]

**Additional Table 1**: **Summary of the clinical data, immunotherapy and sample investigations**

**A)** Multiple different samples from every patient were analyzed in this study using immunohistochemical, flow cytometry (FC), Luminex multiple bead and ELISA techniques. The table summarizes the samples and the investigations. CH/C – chemokine/cytokine levels determination, # special note about the immunotherapy (immunoTx) preceding functional hemispherotomy

| **Patient** | **CSF** | | | | **Blood** | | | | **Tissues** | | | |
| --- | --- | --- | --- | --- | --- | --- | --- | --- | --- | --- | --- | --- |
|  | **prior to immunoTx** | **on immunoTx** | **CH/C** | **FC** | **prior to immuno Tx** | **on immunoTx** | **CH/C** | **FC** | **Immunohistochemistry** | **FC** | **Steroids prior to surgery** | **immunoTx prior to surgery NOTE#** |
| **P1** | 1 | 13 | 14 | 14 | 1 | 19 | 16 | 17 | 10 | 5 | yes | last i.th.MTX 10w |
| **P2** | 0 | 4 | 3 | 2 | 0 | 2 | 2 | 2 | 7 | 3 | yes | last NAT 4w |
| **P3** | 1 | 1 | 2 | 2 | 1 | 1 | 2 | 2 | 8 | 5 | yes | last CPA 60w |
| **P4** | 0 | 1 | 1 | 1 | 0 | 1 | 1 | 1 | 5 | 6 | yes | last CPA 8w |
| **P5** | 0 | 2 | 2 | 2 | 0 | 2 | 2 | 2 | 5 | 3 | yes | i.th.MTX 1w |
| **P6** | 0 | 1 | 1 | 1 | 0 | 1 | 1 | 1 | 6 | 3 | yes | AZA |
| **P7** | 0 | 0 | 0 | 0 | 0 | 1 | 1 | 1 | 4 | 3 | yes | none |
| **Total** | **2** | **22** | **23** | **22** | **2** | **27** | **25** | **26** | **45** | **28** | **all** |  |

**B)** Demographic and clinical data, including immunotherapy (immunoTx), are summarized in the table. All patients manifested with a focal epileptic seizure of individual semiology (median age 4 years, range 2-9; 71% females); preceding infection and/or fever was documented in three cases (P1, P3, P6). In addition, P5 presented with tics and P6 with cerebellar syndrome, and both symptoms were temporal. After an individual prodromal period that characterized infrequent seizures and no hemiparesis, all children developed intractable epilepsy. Time to the correct diagnosis and immunotherapy was delayed since the first disease symptom; three patients received immunotherapy on an empiric basis before the diagnosis.

| **Patient** | **Hmsf.** | **Prodroms** | **Time 1st symptom (months)** | | | **ImmunoTx - overwiev** | | | | | | | |
| --- | --- | --- | --- | --- | --- | --- | --- | --- | --- | --- | --- | --- | --- |
|  |  | **(months)** | **to immunoTx** | **to dg.** | **to surgery** | **Steroids** | **IVIG** | **AZA/TAC/MMF** | **CPA** | **RTX** | **NAT** | **ALEM** | **ITMTX** |
| **P1** | **D** | **3** | 12 | 12 | 44 | **yes** | **yes** | **yes** | no | **yes** | no | **yes** | **yes (8x)** |
| **P2** | **D** | **2** | 5 | 7 | 26 | **yes** | **yes** | no | **yes (4x)** | no | **yes** | no | no |
| **P3** | ND | 1 | 2 | 1 | 22 | **yes** | **yes** | no | **yes (4x)** | no | no | no | no |
| **P4** | ND | 5 | 5 | 5 | 12 | **yes** | **yes** | no | **yes (4x)** | no | no | no | no |
| **P5** | ND | 1 | 3 | 9 | 13 | **yes** | **yes** | **yes** | no | no | no | no | **yes (1x)** |
| **P6** | ND | 0,5 | 3 | 9 | 69 | **yes** | **yes** | **yes** | no | no | no | no | no |
| **P7** | ND | 1 | 115 | 115 | 129 | **yes** | **yes** | no | no | no | no | no | no |

**Abbreviations:** ALEM – alemtuzumab, AZA – azathioprine, CPA – cyclophosphamide, D – dominant hemisphere (hmsf.), ITMTX – intrathecal methotrexate, IVIG – intravenous immunoglobulins, MMF – mycophenolate mophetil, NAT – natalizumab, RTX – rituximab, TAC- tacrolimus

**C)** Patient’s clinical characteristics at three different time points are summarized in the table: 1) modified Rankin Scale (mRS) was used to display the overall disability and dependence, 2) severity of hemiparesis, 3) ability to walk independently (walking), 4) functional use of the affected hand (hand grip), 5) phatic problems (speech) and 6) presence of seizures are described. Intractable epilepsy and individual motor and cognitive decline were the reasons for hemispherotomy in all of our patients. Various types of everyday seizures/ clusters of seizures/ status epilepticus/ epilepsia partialis continua usually represented the intractable epilepsy in our patients; patients with the longest disease duration (P6, P7) had the most pronounced cognitive impairment. Patients with reduced inflammation in their brains after sustainable and aggressive T cells-targeted immunotherapy (P1, P2 and P4) had more favorable mRS score (P1, P4) and mild hemiparesis with preserved functional use of the extremities before hemispherotomy (P1, P2 and P4); hemispherotomy caused aggravation in mRS score in these patient. This contrasted with the remaining ones, who received less aggressive or less intense immunotherapy and had more pronounced inflammation (P3, P5) or degeneration (P6, P7) in their brains at the time of surgery; hemispherotomy caused even improvement of motor functions in some of them (P5). All patients, except the one (P7), remained seizure free after the complete disconnection of the affected hemisphere.

* In P1 alone, the immunotherapy modified the course of epilepsy (periods of relative seizure stabilization alternated clusters of seizures), this was previously described in a brief case report [13]. This patient had no seizures apart from epilepsia partialis continua of the right hand at the time of surgery.

** The recurrence of seizures in P4 was due to the incomplete disconnection of the affected hemisphere; P4 remained seizure free after completing the hemispherotomy.

*** The only one non-seizure free patient (P7) had the longest disease duration, late diagnosis and received minimal immunotherapy before hemispherotomy.

|  | **At diagnosis** | | | | | | **At surgery** | | | | | | **12 months after surgery** | | | | | |
| --- | --- | --- | --- | --- | --- | --- | --- | --- | --- | --- | --- | --- | --- | --- | --- | --- | --- | --- |
|  | **mRS** | **hemi-paresis** | **walking** | **hand grip** | **speech** | **seizures** | **mRS** | **hemi-paresis** | **walking** | **hand grip** | **speech** | **seizures** | **mRS** | **hemi-paresis** | **walking** | **hand grip** | **speech** | **seizures** |
| **P1** | 1 | no | yes | yes | normal | yes | 2 | mild | yes | yes | impaired | yes* | 3 | moderate | yes | no | impaired | no |
| **P2** | 2 | mild | yes | yes | impaired | yes | 3 | mild | yes | yes | impaired | yes | 3 | moderate | yes | no | impaired | no |
| **P3** | 1 | no | yes | yes | normal | yes | 3 | moderate | yes | no | normal | yes | 3 | moderate | yes | no | normal | no |
| **P4** | 1 | mild | yes | yes | normal | yes | 2 | mild | yes | yes | normal | yes | 3 | moderate | yes | no | normal | yes** |
| **P5** | 2 | mild | yes | yes | normal | yes | 5 | moderate | no | no | impaired | yes | 4 | severe | no | no | normal | no |
| **P6** | 2 | mild | yes | yes | normal | yes | 4 | mild | yes | yes | impaired | yes | 4 | severe | no | no | impaired | no |
| **P7** | 4 | moderate | yes | no | impaired | yes | 4 | moderate | yes | no | impaired | yes | 4 | severe | no | no | impaired | yes*** |
