# Supplementary figures and images for: An immunotherapy effect analysis in Rasmussen encephalitis
Source: BMC Neurol. 2020 Sep 24;20:359. doi: 10.1186/s12883-020-01932-9 (PMC7517818; doi:10.1186/s12883-020-01932-9)

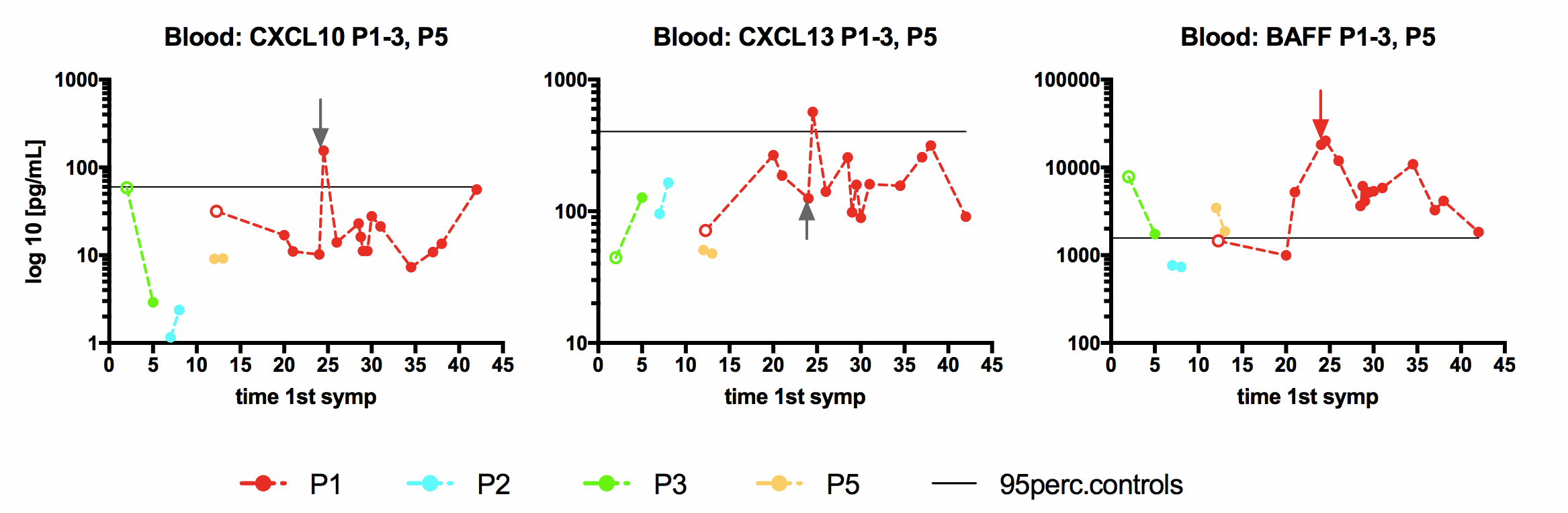

Supplement: Supplementary file 2 — Additional file 2. Individual dynamic changes in chemokine/cytokine levels in the blood during treatment (additional_file_2.tiff). The dynamic changes in the levels of CXCL10, CXCL13 and BAFF levels in the blood are captured. Despite the dynamic changes, the levels of CXCL10 and CXCL13 in the blood generally did not exceed the 95th percentile of controls; this contrasted with the findings in CSF. In contrast, the blood BAFF level frequently exceeded the 95th percentile of controls. In P1, the BAFF level increased after RTX and ALEM treatment and decreased until month 18. In the remaining patients, BAFF levels tended to decrease after treatment.. The patients are indicated with different colors. The empty dots represent samples collected prior to any immunotherapy and the arrows indicate ALEM administration in P1. No blood sample was available from P2 for chemokine/cytokine analysis after the initiation of NAT treatment. [file 12883_2020_1932_MOESM2_ESM.tiff]
